# Analysis of prototypical narratives produced by aphasic individuals and cognitively healthy subjects

**DOI:** 10.1590/1980-57642015DN93000011

**Published:** 2015

**Authors:** Gabriela Silveira, Letícia Lessa Mansur

**Affiliations:** 1 Phonoaudiologist, Department of Physiotherapy, Phonoaudiology and Occupational Therapy of the University of São Paulo School of Medicine.; 2 Associate Professor, Department of Physiotherapy, Phonoaudiology and Occupational Therapy of the University of São Paulo School of Medicine.

**Keywords:** discourse analysis, aphasia, language assessment

## Abstract

Aphasia can globally or selectively affect comprehension and production of verbal and written language. Discourse analysis can aid language assessment and diagnosis.

**Objective:**

[1] To explore narratives that produce a number of valid indicators for diagnosing aphasia in speakers of Brazilian Portuguese.
[2] To analyze the macrostructural aspects of the discourse of normal individuals.
[3] To analyze the macrostructural aspects of the discourse of aphasic individuals.

**Methods:**

The macrostructural aspects of three narratives produced by aphasic individuals and cognitively healthy subjects were analyzed.

**Results:**

A total of 30 volunteers were examined comprising 10 aphasic individuals (AG) and 20 healthy controls (CG). The CG included 5 males. The CG had a mean age of 38.9 years (SD=15.61) and mean schooling of 13 years (SD=2.67) whereas the AG had a mean age of 51.7 years (SD=17.3) and mean schooling of 9.1 years (SD=3.69). Participants were asked to narrate three fairy tales as a basis for analyzing the macrostructure of discourse. Comparison of the three narratives revealed no statistically significant difference in number of propositions produced by the groups. A significant negative correlation was found between age and number of propositions produced. Also, statistically significant differences were observed in the number of propositions produced by the individuals in the CG and the AG for the three tales.

**Conclusion:**

It was concluded that the three tales are applicable for discourse assessment, containing a similar number of propositions and differentiating aphasic individuals and cognitively healthy subjects based on analysis of the macrostructure of discourse.

## Abstract (pt)

A afasia acomete de forma global ou seletiva a compreensão e a produção da linguagem oral e escrita. A análise do discurso pode auxiliar a avaliação e contribuir para o diagnóstico de linguagem.

**Objetivo:**

[1] Explorar narrativas que produzam o número de indicadores válidos para diagnóstico de afasia em sujeitos falantes do Português Brasileiro.
[2] Analisar aspectos macroestruturais do discurso de indivíduos normais.
[3] Analisar aspectos macroestruturais do discurso de indivíduos afásicos.

**Métodos:**

Foram analisados aspectos macroestruturais de três narrativas produzidas por indivíduos afásicos e sadios do ponto de vista cognitivo.

**Resultados:**

Foram examinados 30 voluntários, 10 afásicos (GA) e 20 indivíduos sadios (GC). O GC era composto de 5 indivíduos do gênero masculino. A média de idade desse grupo foi 38,9 (dP=15,61) e a de escolaridade 13 (dP=2,67). A média de idade do GA foi 51,7 (dP=17,3) e de escolaridade foi 9,1 anos (dP=3,69). Os sujeitos foram solicitados a narrar três histórias que serviram de base para análise da macroestrutura do discurso. Não houve diferenças estatisticamente significativas no número de proposições produzidas quando as três histórias foram comparadas. Houve correlação significativa, negativa, entre idade e número de proposições emitidas. Houve diferenças significativas no número de proposições emitidas pelos indivíduos do GC e do GA nas três histórias.

**Conclusão:**

Foi possível concluir que as três histórias são aplicáveis para avaliação de discurso, pois apresentam número similar de proposições e diferenciam afásicos e sujeitos sadios do ponto de vista cognitivo, a partir da análise de macroestrutura do discurso.

## INTRODUCTION

Aphasia can be caused by stroke and present as global or selective impairment in comprehension and production of spoken or written language.^1^

Since the nineteenth century, two major aphasic syndromes have been established: Wernicke's and Broca's aphasias. These syndromes affect two basic language operations: selection and sequencing.^2^

However, the taxonomy of aphasia is somewhat broader, encompassing several other subtypes of aphasia: anomic, motor and sensory transcortical, mixed and global, as well as conditions not included under any specific classification.^3^ Anomia is present in all aphasic disorders but is the predominant and characteristic symptom in anomic aphasia.

Four types of aphasia: Broca's, Wernicke's anomic and mixed, were selected to investigate narrative discourse.

The criteria employed for classifying the aphasias are fluency, auditory comprehension and repetition.^2,3^ Broca's aphasia is classified under the nonfluent conditions. The syndromic description of this type of aphasia includes functional comprehension (except for complex syntactic elements) along with repetition and naming deficits. In severe cases, initial mutism or limited speech and stereotypies may occur. In less severe cases, spontaneous language is nonfluent, exhibiting a syntactically impaired, telegraphic style, limited to nouns (high frequency and imageability), with scant verbs and the presence of phonemic paraphasias. Speech produced is effortful with changes in articulation (dysarthria)^4^ and speech programming difficulties (speech apraxia).^4^ Long pauses and fillers evidence difficulty in accessing words. Individuals with Wernicke's aphasia are fluent, able to produce language effortlessly and exhibit good articulation. However, the content of output is marked by the presence of paraphasias, particularly phonemic. These patients present changes in verbal language comprehension at all levels and have difficulties in repeating.

Assessment of aphasic patients includes observation of preserved and altered aspects of language and speech. Discourse represents an interesting perspective of language analysis, since it allows the observation of various different components of language (phonetic, phonological, morphologic and syntactic), besides the interaction of language with other cognitive aspects such as attention, working memory, episodic memory and executive function.

Discourse can be defined according to different theoretical models. According to the formalist or structuralist view, discourse is a unit of language larger than a sentence, whilst according to the functional view, discourse represents language in use.^5^

Discourse can be analyzed at a micro and macrostructural levels. At the microstructural level, phonologic, lexical-semantic and syntactic aspects are analyzed. At the macrostructural level, global meaning (gist), the organization of meanings in the text, context and purpose of the text from a social perspective are analyzed.^5^ There are two theories explaining the mechanisms of macroprocessing. One theory^7^ holds that macroprocessing generalizes and summarizes the contents of micropropositions; while the second theory^6,8^ advocates that world knowledge and pragmatic reasoning play a pivotal role in the macroprocessing of discourse.

Given that the discourse of aphasic individuals exhibits changes in microstructure, it is possible to observe the impact of such alterations on macrostructure. In the case of greater support on microstructure, the the impact of changes arising in the construction of macrostructure is higher. If support on world knowledge or keywords prevails, the impact of aphasia is attenuated. It is also important to bear in mind that, in the different types of aphasia, factors such as fluency cause different changes in microstructure which in turn can affect the constitution of macrostructure differently.

In the area of Neuropsychology, single or sequences of scenes, conversations and biographical accounts are used to study discourse.^5^

The Aphasia Bank^9^ proposes the use of children's stories (fairy tales) supported by figures, for discourse analysis. The Cinderella story was included in the bank for being a familiar fairy tale in Western culture and for generating, in a short timeframe, sufficient material for analysis.^10^ Moreover, the study based on prototypical narratives such as Cinderella yielded data that was easily recognizable by the examiner. In addition, besides allowing analysis of macrostructure, these tales are useful for examining microstructure by sparing mnesic recall and executive effort in organizing the material.^10^

Cinderella is one of the most well-known fairy tales in Brazil, together with Snow White, the Seven Dwarfs and Little Red Riding Hood, although it is not known whether knowledge of the three stories by the Brazilian population is equivalent.

The objectives of the present study were:

[1] To explore children's narratives (fairy tales) that produce a valid number of indicators for studying discourse in a sample of speakers of Brazilian Portuguese.
[2] To analyze the macrostructural aspects of the discourse of normal individuals.
[3] To analyze the macrostructural aspects of the discourse of aphasic individuals.

## METHODS

This study was approved by the Ethics Committee for Analysis of Research Projects of the Clinicas Hospital of the University of São Paulo School of Medicine (HCFMUSP) under number 777.615. All participants signed the Free and Informed Consent Form.

The study involved two groups: a control group of cognitively-healthy volunteers (CG) and an aphasia group comprising patients with vascular stroke-induced aphasia (AG).

The inclusion criteria for the CG were: absence of dementia conditions or uncontrolled comorbidities, absence of behavioral or psychiatric disorders, and no neurologic impairments. The control subjects were recruited randomly from community volunteers. The inclusion criteria for the AG were: be registered in the outpatient language neurologic unit of the Neurologic Medicine Division of the HCFMUSP, absence of dementia or uncontrolled comorbidities, absence of behavioral or psychiatric disorders, and no neurologic impairments except for stroke which induced the aphasia, and having an aphasia severity score >2.^15^This severity measure reflects verbal production ability, including speech apraxia and dysarthria. A cut-off point of intelligibility of speech >5 was defined for inclusion.^4^ The CG was submitted to screening assessment to check normality criteria: the Mini-Mental State Exam;^11^ Verbal Fluency- semantic (animals)^12^ and phonemic criteria^13^ in addition to MOANS criteria.^14^ The AG was thus formed after interpretation based on the Boston Diagnostic Aphasia Examination, which includes a severity score and screening for apraxia and dysarthrias.^15^

The scenes for the fairy tales were illustrated in black and white drawings based on a prototypical version. The participants were asked to examine the scenes of Cinderella, Snow White & the Seven Dwarfs and Little Red Riding Hood, and to tell each story in their own words. The Snow White & the Seven Dwarfs and Little Red Riding Hood tales were included in the study but no information is available on the clinical applicability of these tales as assessment instruments. No time limit was stipulated for speech production and the examiner only intervened to prompt commencement or continuation of the narrative.

The discourse produced was video recorded and the Express Scribe^16^ software was used to aid the transcription process.

Analysis was based on the propositions produced, since these are units of meaning close to the conceptual level of language processing. The propositions of each story were identified based on the prototypical versions taken from Wikipedia. The tales recounted were analyzed separately in a manual fashion. One point was given for each proposition produced in the three tales. The performance of the CG served as a reference for interpreting the results of the AG, since no standardized expected performance norms exist. Analysis of the narratives produced, and the attribution of scores, was done by three raters based on consensus.

The total number of propositions produced was used as the basis for comparison among the narratives. In this analysis, independent, intra-group comparison was performed using the Kruskal Wallis test.

The CG and AG groups were compared for number of propositions produced in each of the tales using the Mann Whitney test.

Spearman's test was used to investigate correlations between total number of propositions produced and sociodemographic factors, such as age and schooling.

## RESULTS

Thirty volunteers took part in the study: 10 aphasic individuals (AG) and 20 healthy controls (CG). The CG included 5 male and 15 female participants. The CG had a mean age of 38.9 years (SD=15.61) and mean schooling of 13 years (SD=2.67) whereas the AG had a mean age of 51.7 years (SD=17.3) and mean schooling of 9.1 years (SD=3.69). The groups did not differ for age (p=0.0646) but there was a difference for schooling (p= 0.0175). The aphasic conditions together with a detailed description of the cases are given in Table 1.

**Table 1:** Sample characteristics of Aphasia Group (AG).

	Age	Schooling	Gender*	Aphasia type and comorbidities	Aphasia severity**	Intelligibility of speech
1	51	15	F	Broca’s aphasia; speech apraxia	2	6
2	55	5	M	Broca’s aphasia; speech apraxia; dysarthria	2	5
3	67	8	F	Anomic aphasia	4	7
4	52	5	M	Broca’s aphasia; speech apraxia; dysarthria	2	6
5	34	13	F	Broca’s aphasia; speech apraxia	4	6
6	60	8	F	Anomic aphasia	4	7
7	71	11	M	Anomic aphasia	4	6
8	18	11	F	Mixed aphasia	3	7
9	38	11	F	Anomic aphasia	5	7
10	71	4	M	Mixed aphasia	3	5

* Gender: F=female; M=male.

** Aphasia severity score: 2=patient able to converse about familiar topics, with assistance; 3=can discuss virtually all problems of everyday life with little or no assistance; 4=no significant limitations in ideas or form of expression; 5=minimal noticeable deficits. Speech intelligibility score- 5=Generally reduced under adverse conditions when content is unrestricted; 6=Sometimes reduced under adverse conditions; 7=Sometimes reduced in adverse situations even when content is restricted; but corrected by reformulation.

Table 2 shows the number of propositions produced by AG patients for each tale.

**Table 2:** Propositions produced by AG.

Subjects	Cinderella (Total 22) N (%)	Little Red Riding Hood (Total 14) N (%)	Snow White (Total 19) N (%)
1	4 (18.18)	2 (13.33)	3 (15.78)
2	0 (0)	3 (20)*	5 (26.31)*
3	5 (21.73)	6 (40)	6 (31.57)*
4	4 (18.18)	4 (26.66)	5 (26.31)*
5	12 (52.17)*	6 (40)	8 (42.10)*
6	14 (60.86)*	6 (40)*	2 (10.52)
7	2 (0.086)	5 (33.33)	2 (10.52)
8	2 (0.086)*	7 (46.66)*	4 (21.05)*
9	7 (30.43)*	8 (53.33)*	6 (31.57)*
10	6 (26.08)	0 (0)	4 (21.05)*

* Individuals producing target propositions central to narrative organization. N: number of propositions produced.

The mean percentage of propositions produced by CG subjects was: 67.75 % - Cinderella; 74.33% - Little Red Riding Hood; 62.89% - Snow White.

Table 3 depicts comparative intragroup and intergroup performance for total propositions produced overall and for each tale by the AG and CG.

**Table 3:** Intra and inter-group comparisons for number of propositions produced.

	CG Mean	AG Mean	P (controls × aphasics)
Total propositions (3 tales)	12.21 (3.68)	6 (2.30)	0.0003*
Cinderella	13.55 (11.95)	5.6 (4.427)	
Snow White	11.95 (11.15)	7.7 (3.4)	
Little Red Riding Hood	11.15 (13.55)	4.7 (2.451)	
p (intra-groups)	0.2466**	0.1875***	

* Comparison of CG × AG for mean number of propositions produced in the three tales. Mann- Whitney Test.

** Comparison of tales (Red Riding Hood × Snow White × Cinderella) for number of propositions produced in CG. Wilcoxon Test.

*** Comparison of tales (Red Riding Hood × Snow White × Cinderella) for number of propositions produced in AG. Wilcoxon Test.

Comparison of the narratives in the CG revealed no differences in total propositions produced for each tale (p=0.2466). This same finding was observed in the AG (p=0.1875) (Table 3).

Potential associations of performance with sociodemographic variables (age and schooling) and lesion (aphasia severity) in the AG group were analyzed (Table 4). A significant negative correlation was found between age and number of propositions produced.

**Table 4:** Correlation of age and schooling with total number of propositions for the three tales (Cinderella + Red Riding Hood + Snow White) in the CG and AG.

Variables		rs	p value
CG Tot propositions	× Age	–0.5403	0.0139
	× Schooling	0.2194	0.3527
	× Age	–0.5030	0.1382
AG Tot propositions	× Schooling	0.1548	0.6693
	× Aphasia Severity	0.5260	0.1183

Tot: total; CG: Control Group; AG: Aphasic Group; rs: Spearman’s correlation coefficient.

Significant differences were detected in number of propositions produced by CG and AG participants for the three tales (Cinderella, p=0.0007; Little Red Riding Hood=0.0002). This difference was lower for the Snow White tale (p=0.0679). In the CG, results showed a negative age effect, whereas in the AG no effects of sociodemographic variables were evident (Table 4).

The transcription time of the discourses was reduced by an average of 4 minutes using the *Express Scribe* software, as compared to conventional transcription times.

## DISCUSSION

The aim of the present study was to analyze the macrostructure of healthy controls and patients with stroke-induced aphasia.

The analysis focused on global meaning (gist) and on the way in which the meanings were organized from a contextual perspective.^5^ Thus, the ability to produce the components structuring the semantic axis of the discourse was observed.

The potential of prototypical narratives illustrated in figurative scenes was exploited as a data collection instrument for analysis of the discourse of healthy and aphasic speakers of Portuguese. We investigated the advantages of retaining the Cinderella fairy tale used in the Aphasia Bank project^9^ or replacing it with other tales for assessing discourse.

The three tales assessed contained a similar number of propositions and therefore can all be used for discourse analyses in clinical settings.

At the macrostructure level, the AG produced a lower number of propositions on the three tales compared to the number produced by the CG.

It is noteworthy that the AG was both small and contained predominantly young individuals with Broca's and anomic aphasias. In patients with Broca's aphasia, agrammatism and speech apraxia, associated with the anomia which occurs in all aphasic conditions, was expected to impair microstructure production as well as elementary aspects of discourse such as macrostructure key words. Given no time limits were imposed for producing the tale, it is believed that the performance of subjects was not affected by speech motor difficulties. On the other hand, one subject with anomic aphasia was among those who produced the fewest propositions for the macrostructure (Subject 3). Andreetta, Cantagallo & Marini^17^ studied the macrostructure of the narrative of chronic anomic aphasia patients and concluded that changes in microstructure impact the cohesion and coherence of macrostructure.

Huber^18^ pointed out that macroprocessing based on knowledge beyond the microstructure may be compromised in aphasic patients, although acknowledged the difficulty in specifying the mechanisms underlying this change: difficulties in propositional reasoning or choosing amidst the high demand of concomitant investments in micro and macrostructure. In the latter case, there are advantages choosing cognitive investment in micro or macrostructure depending on the situation.

If we accept the hypothesis that individuals rely on knowledge beyond the microstructure to produce a macro-structure, one must take into account the possibility that both healthy and aphasic individuals shared knowledge, about this prototypical story with the examiner.

Although sociodemographic variables can influence the organization of discourse, disparities in age and schooling between the control and aphasia groups did not affect results for the AG since there was no association of proposition production with age and schooling in the group. The effect of age on the number of propositions noted in the control group was previously explained by other researchers as a preference of older individuals for a style that emphasizes the semantic axis of the story.^19^ This finding warrants further studies.

The severity of aphasia should be highlighted in that the present sample comprised relatively young subjects with chronic, moderate-to-severe aphasia. Among these subjects, the severity of language impairment can predominate over other cognitive aspects (such as executive) which may be associated with aphasia particularly in older adults.^20^

Strengths of the present study include the use of a transcription instrument, significantly reducing the time required for this task. The use of this tool is congruent with the need to build a bank holding representative data on aphasic and healthy individuals.

Study limitations include the small sample size for both AG and CG as well as the exclusion of microstructure data from the analysis. Future studies could explore correlations between macro and microstructure measurements to identify possible associations between these processes.

Despite the limitations of this study, it can be concluded that the three tales contain a similar number of propositions, indicating their equivalence as data collection instruments for analyzing the discourse of speakers of Brazilian Portuguese. In addition, the macrostructure analysis cognitively differentiated aphasic patients from healthy subjects. Larger samples are needed to obtain reliable references for sociodemographic variables.
